# Post-Harvest Induced Production of Salvianolic Acids and Significant Promotion of Antioxidant Properties in Roots of *Salvia miltiorrhiza* (Danshen)

**DOI:** 10.3390/molecules19067207

**Published:** 2014-05-30

**Authors:** Guo-Jun Zhou, Wei Wang, Xiao-Mei Xie, Min-Jian Qin, Ben-Ke Kuai, Tong-Shui Zhou

**Affiliations:** 1 Research Center of Natural Products, Institute of Plant Biology, Fudan University, Shanghai 200433, China; 2 Department of Resources Science of Traditional Chinese Medicines & Key Laboratory of Modern Chinese Medicines (Ministry of Education), China Pharmaceutical University, Nanjing 210009, China; 3 School of Traditional Chinese Pharmacy, Anhui University of Chinese Medicine, Hefei 230038, China

**Keywords:** accumulation after harvest, dehydration inducement, radical scavenging activity, *Salvia miltiorrhiza*, salvianolic acid B

## Abstract

Danshen, the dried roots of *Salvia miltiorrhiza*, is an extremely valued Traditional Chinese Medicine. Previously, we have demonstrated that salvianolic acid B (SaB), the important bioactive ingredient in this herb, was a post-harvest product. Here, we further reported that all salvianolic acids (SAs) in the roots were post-harvest products of the drying process. In addition, the results of various radical scavenging activity assays, including lipid peroxidation (1), DPPH (2), hydroxyl (3) and superoxide (4), were significantly increased along with the accumulation of total salvianolic acids in the process. The contents of chemical targets and antioxidant activities both reached the highest value under thermal treatment at 130 °C for 80 min. In this dehydration period, contents of SaB, and sum of nine SAs increased from 0.01% to 5.51%, and 0.20% to 6.61%; and IC_50_ of antioxidant activity decreased from 4.85 to 2.69 (1); 7.75 to 0.43 (2); 2.57 to 1.13 (3) and 17.25 to 1.10 mg/mL. These results further supported the hypothesis that the newly harvested plant roots were still physiologically active and the secondary metabolites might be produced due to dehydration stress after harvest. Our findings supplied an important and useful theoretical basis for promoting the quality of Danshen and other medicinal plant materials.

## 1. Introduction

**Figure 1:**
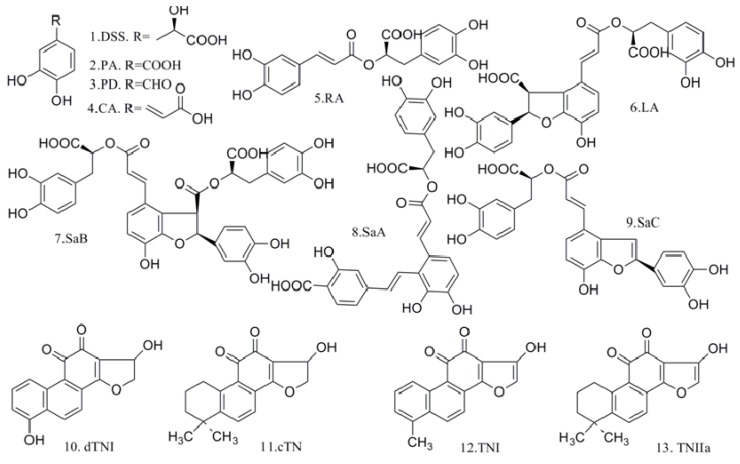
Chemical structures of the major bioactive components in Danshen. (1) Danshensu (DSS); (2) protocatechuic acid (PA); (3) protocatechualdehyde (PD); (4) caffeic acid (CA); (5) rosmarinic acid (RA); (6) lithospermic acid (LA); (7) salvianolic acid B (SaB); (8) salvianolic acid A (SaA); (9) salvianolic acid C (SaC); (10) dihydrotanshinone I(dTNI); (11) cryptotanshinone (cTN); (12) tanshinone I (TNI); (13) tanshinone IIa (TNIIa).

Danshen, the dried roots of *Salvia miltiorrhiza* Bge. (Labiatae), is one of the most important and highly valued Traditional Chinese Medicines which is currently receiving worldwide attention for its potential to prevent and treat cardiovascular diseases [1,2,3]. Numerous Danshen preparations, in the form of tablets, capsules, granules, injections, oral liquids, dripping pills, or sprays, made from either Danshen alone or in combination with other herbal ingredients (Fufang in Chinese), are commercially available in China and other countries [2,4]. Phytochemical and pharmacological investigations have revealed that both salvianolic acids (SAs) and tanshinones (TNs) are responsible for its bioactive effects [2,5,6,7]. The water-soluble SAs mainly include danshensu (DSS), protocatechuic acid (PA), protocatechualdehyde (PD), caffeic aid (CA), rosmarinic acid (RA), lithospermic acid (LA), salvianolic acid B (SaB), salvianolic acid A (SaA), and salvianolic acid C (SaC). The TNs are lipid-soluble and mainly composed of dihydrotanshinone I (dTNI), cryptotanshinone (cTN), tanshinone I (TNI), and tanshinone II_A_ (TNII_a_) (Figure 1).

The commercial products of this herb are standardized by content of SaB (≥3.0%) and TNII_a_ (≥0.2%) according to the 2010 Chinese Pharmacopoeia [8]. Thus far, a number of publications have evaluated the quality of Danshen and its preparations [9]. These publications documented that levels of SaB, TNII_a_, and other ingredients in *S. miltiorrhiza* roots and their preparations varied significantly [9,10,11]. These variations were usually ascribed to differences in germplasm and environmental/climate factors [12] or to the sensitivity of these compounds to light and temperature [13].

Drying is the most common method for the post-harvest processing of medicinal plants [14,15]. The general opinion is that levels of secondary metabolites in plants were accumulated during growth and were decreased in the post-harvest process [16,17]. Therefore, many researchers have focused on how to retain the initial levels and believed that the freeze-drying method was the most suitable method [18]. However, newly harvested plant materials, especially the roots, are still physiologically active organs and the anti-dehydration mechanisms of plants will be induced during the early stage of the dewatering process [19,20]. Various studies have demonstrated that the antioxidant activities of plants could be enhanced during post-harvest processing through the generation of endogenous antioxidant enzymes or secondary metabolites [21,22,23].

Our previous study reported that SaB, the most important and abundant bioactive component of Danshen, was actually a product of the post-harvest drying process at 50–120 °C oven temperature; while the compositions and contents of all TNs were almost unchanged [24]. However, the SaB content still increased even at 120 °C [24]. The most suitable temperature and the inflection point for production of SaB were still obscure. In addition, the change trends of other SAs aside from SaB and the overall antioxidant activities (OAs) needed to be comprehensively explored. These messages are important for verifying our previous findings and uncovering the biosynthetic mechanism and the possible precursor of SaB production.

In this study, we continued to evaluate the effects of thermal processes at higher oven temperatures from 60 to 150 °C on the dynamic variation of SaB and other major SAs. After determining the most suitable temperature, *i.e.*, 130 °C, we conducted a comprehensive analysis on changes of SAs and OAs during the dehydration process.

## 2. Results and Discussion

Two independent experiments were conducted in 2008 (T1) and 2010 (T2), respectively. In T1, the drying temperature ranged from 60–150 °C. The results revealed that 130 °C was the optimal temperature for the production of SAs. Therefore, we designed T2 using 130 °C for further studies on SAs and OAs in the process.

### 2.1. Dehydration Curves

**Figure 2:**
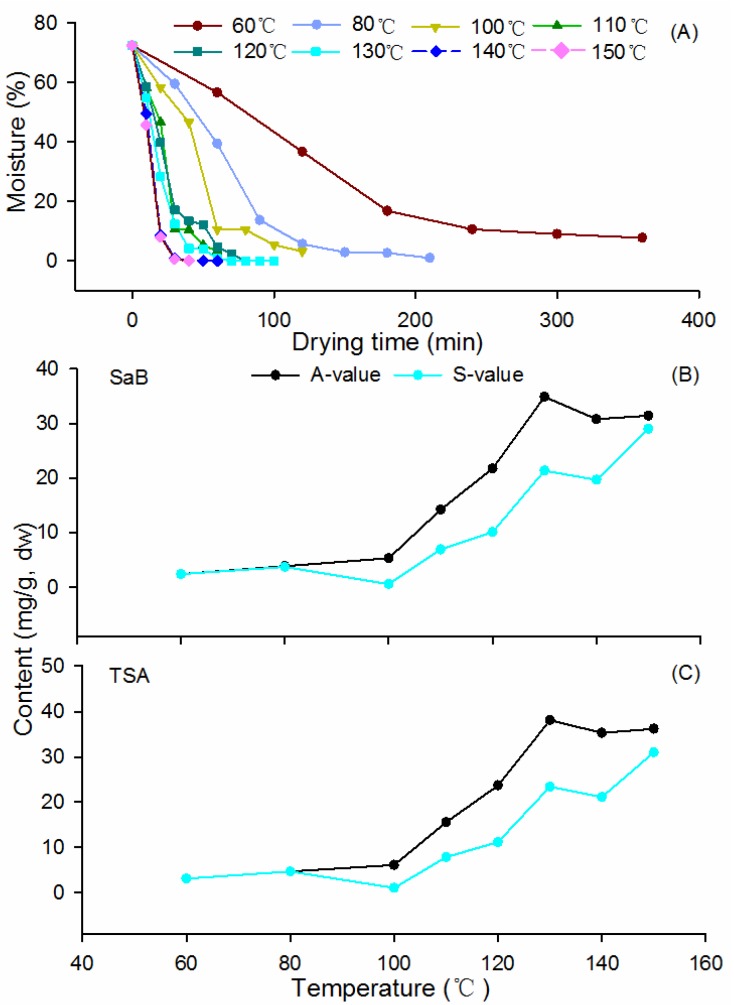
Plots of (**A**) sample moisture at different drying temperatures and (**B**) contents of salvianolic acid B and (**C**) sums of nine salvianolic acidscontents in Danshen dried at 130 °C as a function of temperature and drying time. S-value: content in sufficiently dried samples (moisture ≤ 13.0%); A-value: maximal contents during drying.

The dehydration curves of fresh samples at different temperatures in T1 are illustrated in Figure 2A. Times required for the fresh roots (70% moisture) to dry to the standard moisture level (<13%) at 60–130 °C were 240, 90, 60, 60, 40, 30 min, respectively. Statistical analysis indicated the dewatering efficacy was significantly temperature dependent (*p* < 0.01), and best fitted a nonlinear quadratic polynomial model with the regression equation: Y = aX^2^ − bX + c (R^2^ > 0.95) (where Y is moisture; X is temperature; a, b and c are constants that vary with temperature; Supplementary Material Table S9). A significant positive correlation between dewater rate and oven temperature was observed (*p* < 0.01, data not shown). Oven temperatures at 140–150 °C were too high, which resulted in over-drying and a great loss of SAs from the roots.

Traditionally, the newly harvested Danshen roots were dried under the sun and the process would take about one month. The moisture level was the sole criterion for process and products. The long dehydration duration might result in a marked change of bioactive ingredients of the roots, while reports on its pattern were still absent in the literature up to now. Oven drying might be a promising technique to shorten the duration of dewatering and to promote product quality. Our results provide a useful attempt for optimizing the post-harvest processing method for Danshen production.

### 2.2. Determination of SAs

#### 2.2.1. HPLC Analysis of SAs

**Figure 3:**
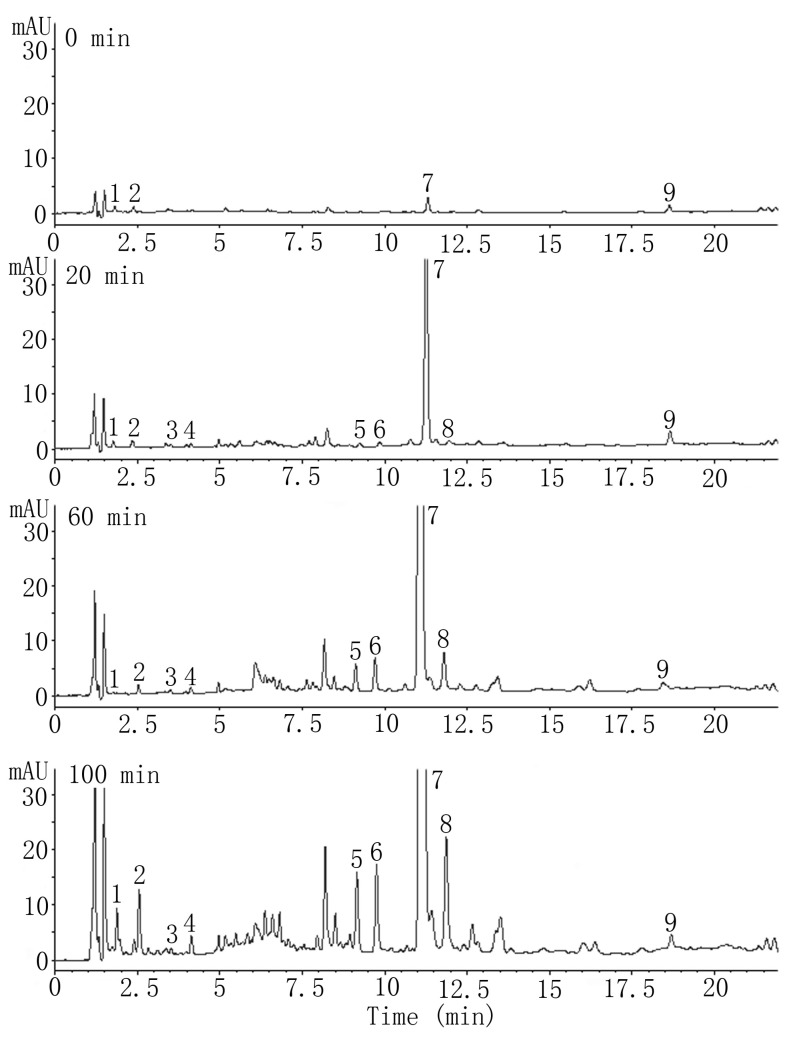
Typical chromatograms of salvianolic acid constituents in Danshen samples dried for different durations at 130 °C. Peak numbers represent: (1) DSS; (2) PA; (3) PD; (4) CA; (5) RA; (6) LA; (7) SaB; (8) SaA; and (9) SaC. See Figure 1 for analyte abbreviations.

Numerous Danshen sample preparation procedures using methanol, water, and 10%–90% aqueous methanol (aM) as extraction solvents have been investigated. The most suitable solvent was proven to be 70% aM; thus it was widely applied to evaluate the quality of Danshen and its preparations [25,26,27]. However, we discovered that an additional water extraction was necessary for maximal assessment of SAs, especially SaC. HPLC chromatograms of samples after different drying durations at 130 °C are shown in Figure 3. The results showed that all SAs appeared gradually during the drying process. Additionally, no distinct SAs peaks were seen in the fresh tissues or before the emergence of SaB, suggesting that other SAs might not be the precursors of SaB.

The regression equations and test ranges for each analyte are shown in Table 1. All calibration curves showed good linear regression (*r^2^* > 0.999) within the test ranges. The recoveries of nine investigated components were 96.9%–104.4%. The results indicated that our new approach for evaluating contents of Danshen SAs was sensitive, precise, and accurate.

**Table 1:** Results of calibration curve and method validation for each analyte *^a^*.

Analytes *^b^*	RT	Standard Curve	TR	LOD	LOQ	RPD	STD
1. DSS	2.42	Y = 6.41x + 1.38	0.92–230	0.12	0.39	1.40	1.99
2. PA	3.04	Y = 15.38x + 9.94	0.52–130	0.07	0.23	2.03	1.75
3. PD	3.91	Y = 39.14x + 34.21	0.52–130	0.03	0.11	4.39	4.34
4. CA	5.08	Y = 11.33x + 13.37	1.16–145	0.06	0.19	3.81	4.71
5. RA	10.62	Y = 3.62x + 4.35	1.60–400	0.25	0.83	1.69	4.63
6. LA	11.68	Y = 3.85x + 4.42	1.64–410	0.27	0.92	1.15	3.63
7. SaB	13.73	Y = 4.71x + 8.70	3.40–850	0.35	1.18	1.87	1.45
8. SaA	15.92	Y = 16.0x + 13.26	1.36–340	0.09	0.29	4.74	2.66
9. SaC	20.87	Y = 1.95x − 1.65	2.64–330	0.61	2.02	4.10	4.16

*^a^* Abbreviations and Units: RT: Retention time (min); TR: Test range (μg/mL); LOD: Limit of detection (μg/mL); LOQ: Limit of quantification (μg/mL); RPD: Repeatability (%); STD: Stability (%); *^b^* Abbreviation for each analyte refers to Figure 1.

#### 2.2.2. Production of SAs

**Figure 4:**
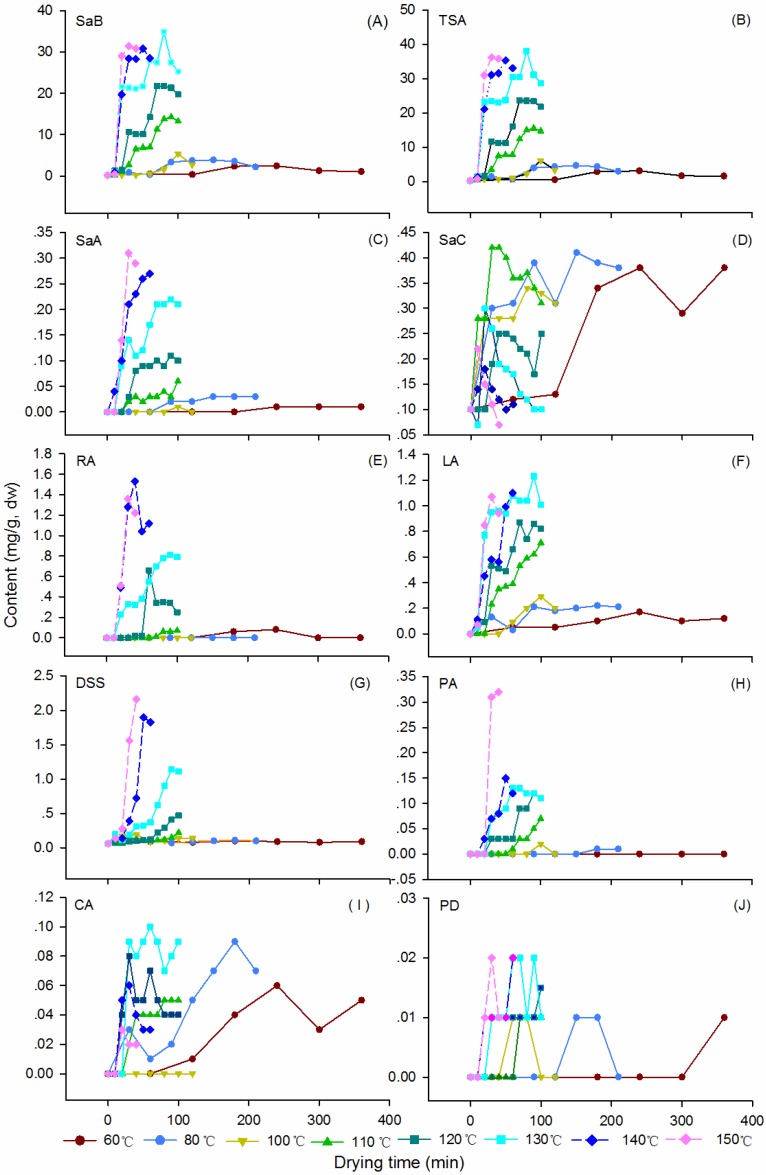
Plots of contents of nine different salvianolic acids in Danshen as a function of drying temperatures and durations. See Figure 1 for abbreviations of analytes. TSA: the summed values of the nine targets.

The changing patterns of nine major SAs during the dehydration process at the designed temperature were illustrated in Figure 4. Detailed data of the assay are shown in Supplementary Material S1–S8. Consistent with our previous report [24], the result demonstrated again that SaB, the most abundant SA in Danshen, was a product of the post-harvest drying process (Figure 4A). The average SaB content in fresh materials was 0.16 mg/g and it increased markedly after dehydration. The production of this compound was not obvious at oven temperatures 60–100 °C, while a rapid and sharp increase was observed at higher temperatures (110–150 °C). The highest level of SaB was found at 130 °C for 80 min. Over the process of dehydration, the contents of SaB showed reverse V-shaped curves and displayed a significant negative correlation (*p* < 0.01) with moisture in the up-curve (Figure 2A and Figure 4A). This level decreased distinctly with prolonged dehydration after the apex, and the rate of decrease was significantly positively correlated with temperature (*p* < 0.05) (Figure 4A).

All other SAs analyzed were also found to be post-harvest products and emerged following the appearance of SaB after dehydration (Figure 4C–J). They were almost undetectable in the fresh roots, however, their contents increased gradually after dewatering at 60–130 °C. The production rates and final yields of these compounds were also temperature dependent. Like SaB, levels of all analytes showed reverse V-shaped curves and displayed significant negative correlations (*p* < 0.01) with moisture in the earlier stages of drying. Total amounts of nine SAs (TSA) changed similarly (Figure 4B). The maximum values of most SAs and TSA were also observed at 130 °C; therefore, this temperature could be regarded as the optimized temperature for the thermal drying process of this herb.

**Figure 5:**
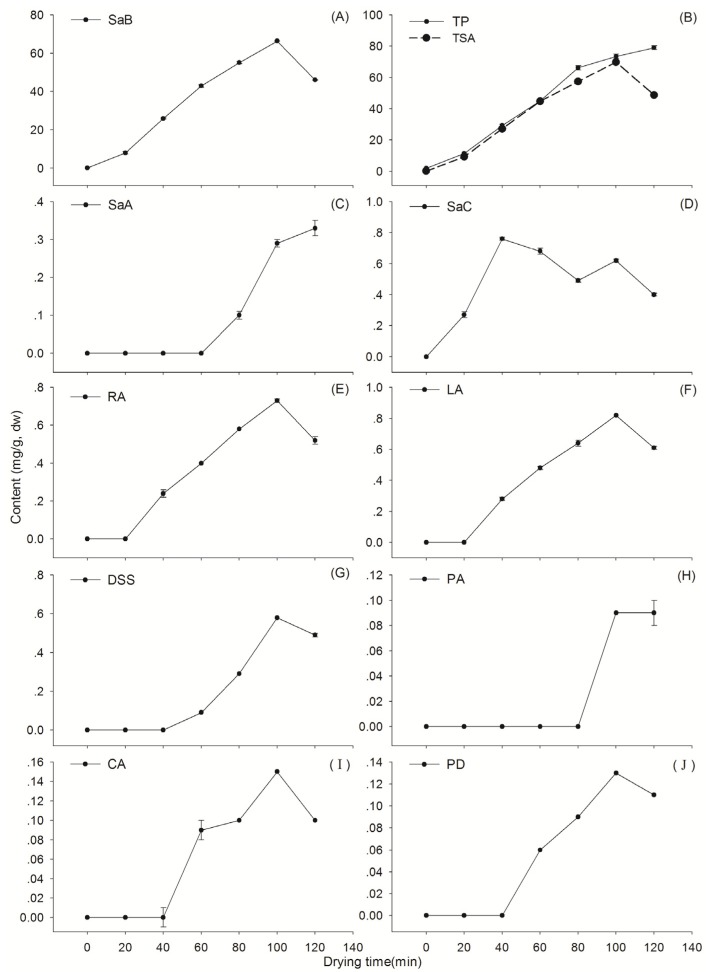
Plots of contents of nine different salvianolic acids in Danshen as a function of drying duration at 130 °C. See Figure 1 for abbreviations of analytes. TSA: summed values of the nine determined targets; TP: total values of phenolic acids determined by colorimetric method.

The above results were further confirmed by T2 (Figure 5). In addition, it showed that the production of other SAs (except SaC) was delayed by at least 40 min after SaB when dried at 130 °C, suggesting that these compounds might be degradation products of SaB. This suggestion was consistent with Xu’s study [28]. To verify the comprehensiveness of HPLC analysis, we carried out a colorimetric detection on the amount of total phenolic acids (TP). Results revealed that the TP value was only slightly higher than the TSA value of the same sample, indicating that the determined targets of HPLC comprised almost all of the SAs in the Danshen roots (Figure 5B).

Levels of SaB and TSA in sufficiently dried samples when moisture ≤ 13.0% (S-values) and their maximum values observed in the drying process (A-values) increased distinctly along with the elevation of drying temperature in the range of 60–130 °C, showing a significant positive correlation (*p* < 0.01) as well. The S-values of both SaB and TSA were markedly lower than their A-values during the drying process at 100–130 °C (Figure 2B,C). In addition, their A-values at each temperature usually occurred earlier than their corresponding S-values. These results told us that when the water level was less than 13%, the roots were actually over-dehydrated from the view of SAs contents. Thus, the prolonged dehydration process resulted in a great loss of bioactive components.

The production mechanism and precursor(s) for the post-harvest production of abundant SaB was a main focus of this study. Unfortunately, we did not find any immediate precursor corresponding to the formation of SaB in fresh materials and during the drying process. Especially, the production of RA was delayed about 40 min after SaB, therefore, we do not agree with the suggestion that RA may be the biosynthetic precursor of SaB as reported in some publications [29,30]. The post-harvest production of SAs might result from remobilization and transformation of pre-harvest stored carbohydrates in roots by a series of primary and secondary metabolic reactions under dehydration stress. This hypothesis needs to be explored in the future.

### 2.3. Promotion of OAs

**Figure 6:**
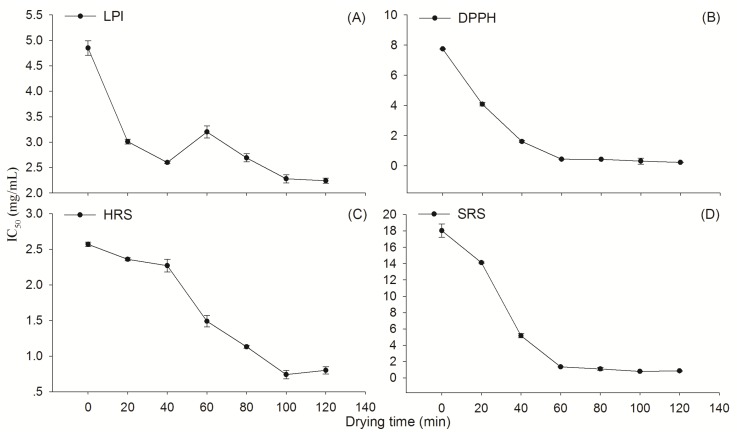
Plots of antioxidant activities IC_50_ in Danshen as a function of drying time at 130 °C. LPI: lipid peroxidation inhibition activity; DPPHs: DPPH radical scavenging activity; HRS: hydroxyl radical (•OH) scavenging activity; SRS: superoxide radical (O^2•–^) scavenging activity.

ROS formation and accumulation is believed to be one of the important causes of many diseases and aging [31,32,33]. The cardiovascular prevention and treating function of Danshen is ascribed to SAs, especially SaB and RA, which possess higher OAs [31,34]. Therefore, a significant promotion of OAs should be the concomitant result with the production SAs during the process of drying. In T2, we tested various OAs including lipid peroxidation inhibition activity (LPI), DPPH radical scavenging assay (DPPHs), hydroxyl scavenging activity (HRS), and superoxide radical scavenging activity (SRS) in 70% aM extracts of dehydrated samples. The IC_50_ values of OAs were generated by a regression analysis of data from a dilution series of extraction solution (Supplementary Materials, Table S11–14). Results showed that levels of various IC_50_ in all extracts decreased significantly along with the procedure of dewater process (Figure 6A–D). The initial value of LPI, DPPHs, HRS, and SRS in the fresh sample was 4.85, 7.75, 2.57, and 17.25 mg/mL, respectively. After 120 min at 130 °C the corresponding value was reduced to 2.24, 0.23, 0.80, and 0.86 mg/mL, decreased by 53.81-, 97.03-, 68.87-, and 95.01-fold. The decreases of OAs IC_50_ exhibited a significant time-dependency (*p* < 0.05) (Supplementary Materials Tables S11–14). Synthesizing the results of our previous report [24] and the present study, we concluded that the significant promotion of OAs was attributed to the production of total SAs during the thermal process. This result supported the idea in some published reports that thermal processing would enhanced OAs by the generation of new compounds or the release of bound phenolic compounds from the breakdown of cellular constituents [21,22,23].

### 2.4. Correlation Analysis

**Figure 7:**
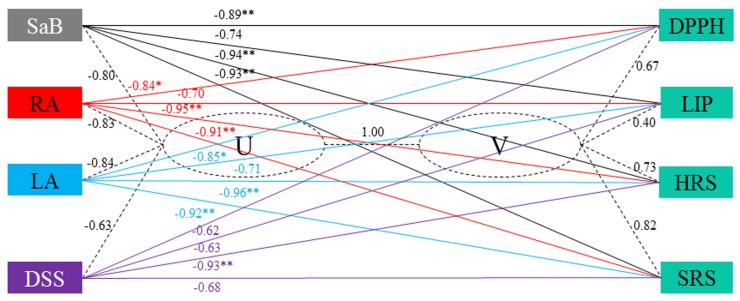
Canonical correlation analysis between contents of four major salvianolic acids and IC_50_ of four antioxidant activities in Danshen samples dried at 130 °C. U: the raw variables of contents; V: the raw variables of IC_50_. Numbers on the black, red, green or purple are coefficient between variables. See Figure 1 and Figure 6 for abbreviations of analytes and antioxidant activities, respectively.

In order to analyze the correlation between SAs levels and OAs IC_50_, bivariate and canonical correlation analyses were applied in T2. To simplify the analysis and to highlight the key ingredients, we selected only values of SaB, RA, LA, and DSS which together accounted for more than 98% of TSA, for the correlation analysis. A significant canonical correlation (1.0, *p* < 0.01) between contents of SAs (U) and IC_50_ of OAs (V) is shown in Figure 7. The canonical loadings in each variable set were above 0.3 with the redundancy index in each set accounted for 0.642 (U) and 0.456 (V), suggesting the effectiveness of the analysis. The correlation coefficients between the raw variables (U and V) and canonical correlation variables (U or V) were also calculated (dotted line in Figure 7). Significant negative correlations between contents of SaB, RA, and LA and IC_50_ values of DPPHs, HRS, and SRS were found (−0.84 to −0.96, *p* < 0.01). The results indicated that these compounds have a strong activity to scavenge DPPH, superoxide and hydroxyl radicals, whereas they have minor function to LIP (−0.70 to −0.74, *p* > 0.05). Notably, DSS had no significant correlations with DPPH, LIP and SRS (−0.62 to −0.68, *p* > 0.05), but had a significant negative correlation with HRS (−0.93, *p* < 0.01), suggesting its specific quenching effect on HRS. These results were in accordance with another study [35], and might be ascribed to the great difference in solubility of these constituents in water and lipid. Our findings also demonstrated that any single assay for evaluating OAs was incomplete to plant materials, and thus two or more methods should be applied in the analysis [36,37].

## 3. Experimental

### 3.1. Plant Materials, Reagents, and Media

Fresh roots of Danshen were collected from Linqu County, Shandong Province of China, during the harvest season from November 1 to 15 of 2008 (test 1, T1) and 2010 (test 2, T2). Fresh materials were taken to the laboratory within 2 days after harvest and were sliced into 2–3 mm pieces, mixed well, and stored at −20 °C until assay. All samples were authenticated and the voucher specimens were deposited in the Herbarium of Fudan University, Shanghai, China. We purchased the following chemicals as indicated: DSS, PA, PD, CA, RA, LA, SaB, SaA, SaC, and gallic acid from Shanghai Usea (Shanghai, China); 2,2-diphenyl-1-picryhydrazyl (DPPH), *β*-carotene, linoleic acid, ethylene diamine tetraacetic acid (EDTA), xanthine, xanthine oxidase, nitroblue tetrazolium (NBT), and thiobarbituric acid (TBA) from Sigma-Aldrich (Shanghai, China); Folin–Ciocalteu reagent from Solarbio Science & Technology (Beijing, China); and liquid chromatography (LC) grade acetonitrile and trifluoroacetic acid (TFA) from J & K Chemical (Shanghai, China). All other reagents were analytical grade from local companies.

### 3.2. Drying Process

Frozen sliced materials were dried in a thermostatic oven as previously described. For T1, groups of samples were dried at 60, 80, 100, 110, 120, 130, 140, or 150 °C. Frozen material was evenly spread into ⌀ 9 cm watch glasses (30.0 g of material each) and placed in an oven preheated to the appropriate temperature. The number of watch glasses for each test group was determined by the sampling frequency described below. At each sampling point (see below), six watch glasses were randomly extracted from the oven, placed in a desiccator to cool, then each sample was pulverized (80 mesh) and weighed. Three samples were used to determine moisture content and three to determine analyte content. The sampling times at each drying temperature were determined in terms of the variations of drying efficiency at each temperature, *i.e.*, every 1 h for 6 h at 60 °C; every 30 min for 210 min at 80 °C; every 20 min for 120 min at 80 °C; every 10 min for 100 min at 110–130 °C; every 10 min for 60 min at 140 °C; and every 10 min for 40 min at 150 °C. Based on the results of T1, we selected an oven temperature of 130 °C and a sampling interval of 20 min over 2 h for T2. Two triplicate batches at each sampling point were used to analyze (1) moisture and (2) levels of SAs, TP and OAs.

### 3.3. Determination of Moisture

Sample moisture was determined in triplicate by Method I as described in the 2010 Chinese Pharmacopoeia [8]. Accurately weighed powdered samples were dried at 105 °C in an oven until constant weight (at least 5 h). After cooling in a desiccator, samples were reweighed and water loss was calculated.

### 3.4. HPLC Analysis of SAs

The contents of nine SAs in samples were determined by high performance liquid chromatography (HPLC) using an Agilent Series 1100 LC instrument (Agilent Technologies, Santa Clara, CA, USA) equipped with an on-line degasser, a quaternary pump, a diode-array detector, a Kinetex™ C18 column (100 mm × 4.6 mm, 2.6 μm) (Phenomenex, Torrance, CA, USA), and a 5 µL sample loop manual injector. The mobile phase consisted of solvents A (0.1% aqueous TFA (aTFA v/v) and B (acetonitrile). The gradient program was: 7%–18% B at 0–4 min, 18%–24% B at 4–15 min, 24%–33% B at 15–20 min, and 33%–52% B at 20–28 min. The flow rate was 1.0 mL/min and the injection volume was 5 μL. The column temperature was maintained at 30 °C. Re-equilibration between individual runs lasted 10 min. The diode-array detector was set at 280 nm. Each sample was analyzed in triplicate.

A standard stock solution containing DSS (2.3 mg), PA (1.3 mg), PD (1.3 mg), CA (2.9 mg), RA (4.0 mg), LA (4.1 mg), SaB (8.5 mg), SaA (3.4 mg), and SaC (3.3 mg) was dissolved in 70% aM in a total volume of 10 mL. The stock solution was diluted with 70% aM as appropriate for calibration and method validation.

Analyte contents were determined as follows: powdered samples (~1.0 g) were extracted twice (20 min each) with 20 mL 70% aM and once with 10 mL water in an ultrasonic bath at room temperature. After centrifugation (1750 *×g*, 10 min), the supernatants were combined and diluted with 70% aM to 50 mL. Some (0.5 mL) of the solution was filtered through a 0.22 μm nylon syringe filter (Millex-HN, Millipore, Billerica, MA, USA) for HPLC analysis. Amounts of each analyte were determined by regression analysis and calculated per unit of sample dry weight (DW) by deducting moisture content. Triplicate analyses were conducted for each sampling point. The remainder of each sample solution was vacuum dried and the residue was dissolved in 50 mL 70% aM to analyze TP and OAs.

### 3.5. Assessment of TP

Levels of TP in samples were determined using a modified colorimetric Folin-Ciocalteu method [37]. Sample solutions (200 μL) were mixed with 200 μL of Folin-Ciocalteu reagent and incubated in a 25 °C water bath for 3 min; 5 mL of 3% Na_2_CO_3_ solution were then added to the mixture. After 30 min, the absorbance at 760 nm was measured. Results were expressed as gallic acid equivalents.

### 3.6. Assay of OAs

*LIP:* LIP was detected using the β-carotene bleaching method of Chan *et al*. with modification [38]. Briefly, 35 mg of linoleic acid and 380 mg of Tween 40 were added to 3 mL of β*-*carotene (4.7 mg in 50 mL chloroform) and mixed thoroughly. The β-carotene/linoleic acid emulsion was prepared by evaporating the chloroform under reduced pressure and adding 100 mL oxygenated ultra-pure water. Initial absorbance of the emulsion (A_initial_) was measured at 470 nm. Aliquots (3 mL) of the emulsion were mixed with 200 μL of a sample solution and incubated in a water bath at 50 °C for 1 h. Absorbance (A_sample_) was measured at 470 nm, and LIP activity was calculated as follows:

LIP (%) = (1 − A_initial_/A_sample_) × 100 (1)

IC_50_ values were generated by regression analysis of data from a dilution series of extraction solutions.

*DPPHs:* DPPHs was analyzed as reported by Chan *et al.* with minor modifications [38]. Briefly, 200 μL of the sample solution was added into 3 mL of DPPH solution (2.1 mg/mL in methanol). The mixture was shaken vigorously and incubated at room temperature in the dark for 30 min. Absorbance of samples (A_sample_) and blanks (A_blank_; using extract solvent instead of sample solution) was measured at 517 nm. DPPHs activity was calculated as follows:

DPPHs (%) = (1 − A_sample_/A_blank_) × 100 (2)

IC_50_ values were generated by regression analysis of data from a dilution series of extraction solutions.

*HRS:* HRS was determined using the Fenton assay according to the method of Halliwell *et al*. with slight modifications [39]. Reagents in a final volume of 3.5 mL of reaction mixture were as follows: salicylic acid (12 mM), FeSO_4_·7H_2_O (0.8 mM), EDTA (0.4 mM), potassium phosphate buffer (pH 7.4, 0.08 mM), 24 mM H_2_O_2_, and 0.5 mL of sample solution. The mixtures were incubated for 2 h at 37 °C. The absorbance of samples (A_sample_) and blanks (A_blank_, the reaction mixture lacking sample solution) was measured at 510 nm against blank. HRS activity was calculated as follows:

HRS (%) = (1 − A_sample_/A_blank_) × 100 (3)

IC_50_ values were generated by regression analysis of data from a dilution series of extraction solutions.

*SRS:* SRS was measured as reported by Beauchamp *et al*. with some modifications [40]. Reagents in a final reaction volume of 3.0 mL were as follows: xanthine (0.3 mM), xanthine oxidase (0.35 U/mL), NBT (7.5 μM), EDTA (23.6 μM), sodium carbonate–sodium bicarbonate buffer solution (50 mM, pH 10.2), and 0.2 mL of sample solution. The mixtures were incubated for 30 min at 25 °C. Absorbance of samples (A_sample_) and blanks (A_blank_, reaction mixture lacking sample solution) was measured at 560 nm. SRS was calculated as follows:

SRS (%) = (1 − A_sample_/A_blank_) × 100 (4)

IC_50_ values were generated by regression analysis of data from a dilution series of extraction solutions.

### 3.7. Statistical Analyses

The correlation analysis was carried out by the bivariate and the canonical correlation analyzing programs in SPSS 11.5 (IBM, Chicago, IL, USA).

## 4. Conclusions

Results of the present study further demonstrated that SaB, the predominant bioactive constituent of Danshen, was a post-harvest product. Besides, other SAs were also found to be the result of dehydration stress after harvesting. At the same time, the OAs of roots, including LIP, DPPHs, HRS and SRS, were significantly promoted as well. The production of SAs and promotion of OAs reached a plateau after 80 min at 130 °C. No SAs in sufficient quantities could be regarded as the direct precursors for the forming of SaB in the fresh and the dewatered samples, leaving a great query for the future exploration. Our findings elucidated one of the reasons that caused the great variation in the quality of Danshen and provided a reasonable solution to this problem. Our reports also offered an important and useful theoretical basis for enhancing the quality of other medicinal plant materials. The further researches include: (1) the dynamic changes of SAs in Danshen roots dried in the sun or in the shade; (2) the transformation mechanism and precursor(s) for the production of SaB in the dehydration process; and (3) the optimized method for the post-harvest drying procedure for producing high quality Danshen herbal materials. These works are still under investigation in our laboratory.

## Supplementary Materials

Supplementary materials can be accessed at: http://www.mdpi.com/1420-3049/19/6/7207/s1.
