# Supplementary material for: Post-Harvest Induced Production of Salvianolic Acids and Significant Promotion of Antioxidant Properties in Roots of Salvia miltiorrhiza (Danshen)
Source: Molecules. 2014 May 30;19(6):7207–22. doi: 10.3390/molecules19067207 (PMC6271733; doi:10.3390/molecules19067207)
Supplement: Supplementary file 1 [file molecules-19-07207-s001.pdf]

Supplementary Materials

Table S1. Contents of hydrophilic components and moisture in Danshen during drying at 60 ℃ (n = 3).

**Analyte (mg/g)**	**Drying time (min)**						
	**0**	**60**	**120**	**180**	**240**	**300**	**360**
Moisture (%)	72.48 ± 0.84	56.68 ± 0.80	36.77 ± 2.47	16.83 ± 0.81	10.66 ± 0.73	9.04 ± 0.47	7.77 ± 0.57
Danshensu	0.06 ± 0.00	0.09 ± 0.00	0.08 ± 0.00	0.10 ± 0.00	0.09 ± 0.00	0.08 ± 0.00	0.09 ± 0.00
Protocatechuic acid	ND	ND	ND	ND	ND	ND	ND
Protocatechualdehyde	ND	ND	ND	ND	ND	ND	0.01 ± 0.00
Caffeic acid	ND	ND	0.01 ± 0.00	0.04 ± 0.00	0.06 ± 0.01	0.03 ± 0.01	0.05 ± 0.00
Rosmarinic acid	ND	ND	ND	0.06 ± 0.00	0.08 ± 0.00	ND	ND
Lithospermic acid	ND	0.05 ± 0.00	0.05 ± 0.00	0.10 ± 0.012	0.17 ± 0.01	0.10 ± 0.01	0.12 ± 0.00
Salvianolic acid B	0.16 ± 0.01	0.48 ± 0.01	0.35 ± 0.00	2.34 ± 0.04	2.44 ± 0.07	1.29 ± 0.03	1.04 ± 0.02
Salvianolic acid A	ND	ND	ND	ND	0.01 ± 0.00	0.01 ± 0.00	0.01 ± 0.00
Salvianolic acid C	0.10 ± 0.00	0.12 ± 0.00	0.13 ± 0.00	0.34 ± 0.00	0.38 ± 0.00	0.29 ± 0.00	0.38 ± 0.00

ND, not detected.

Table S2. Contents of hydrophilic components and moisture in Danshen during drying at 80 ℃ (n = 3).

**Analyte (mg/g)**	**Drying time (min)**							
	**0**	**30**	**60**	**90**	**120**	**150**	**180**	**210**
Moisture (%)	72.48 ± 0.84	59.56 ± 0.50	39.45 ± 0.17	13.75 ± 3.28	5.66 ± 0.86	2.88 ± 0.99	2.65 ± 0.17	1.02 ± 0.57
Danshensu	0.06 ± 0.00	0.15 ± 0.01	0.10 ± 0.02	0.07 ± 0.00	0.09 ± 0.00	0.10 ± 0.01	0.11 ± 0.01	0.10 ± 0.00
Protocatechuic acid	ND	ND	ND	ND	ND	ND	0.01 ± 0.00	0.01 ± 0.00
Protocatechualdehyde	ND	ND	ND	ND	ND	0.01 ± 0.00	0.01 ± 0.00	ND
Caffeic acid	ND	0.03 ± 0.00	0.01 ± 0.00	0.02 ± 0.00	0.05 ± 0.01	0.07 ± 0.00	0.09 ± 0.00	0.07 ± 0.00
Rosmarinic acid	ND	ND	ND	ND	ND	ND	ND	ND
Lithospermic acid	ND	0.13 ± 0.01	0.03 ± 0.00	0.21 ± 0.00	0.18 ± 0.00	0.20 ± 0.00	0.22 ± 0.00	0.21 ± 0.00
Salvianolic acid B	0.16 ± 0.01	0.85 ± 0.00	0.32 ± 0.00	3.32 ± 0.01	3.73 ± 0.05	3.87 ± 0.05	3.51 ± 0.01	2.19 ± 0.02
Salvianolic acid A	ND	ND	ND	0.02 ± 0.00	0.02 ± 0.00	0.03 ± 0.00	0.03 ± 0.00	0.03 ± 0.00
Salvianolic acid C	0.10 ± 0.00	0.30 ± 0.00	0.31 ± 0.00	0.39 ± 0.01	0.31 ± 0.00	0.41 ± 0.00	0.39 ± 0.00	0.38 ± 0.00

ND, not detected.

Table S3. Contents of hydrophilic components and moisture in Danshen during drying at 100 ℃ (*n* = 3).

Analyte (mg/g)	Drying time (min)						
	**0**	**20**	**40**	**60**	**80**	**100**	**120**
Moisture (%)	72.48 ± 0.84	58.27 ± 0.10	46.65 ± 1.30	10.61 ± 0.88	10.46 ± 0.64	5.37 ± 0.26	3.21 ± 0.12
Danshensu	0.06 ± 0.00	0.17 ± 0.00	0.19 ± 0.00	0.09 ± 0.00	0.11 ± 0.01	0.14 ± 0.00	0.14 ± 0.00
Protocatechuic acid	ND	ND	ND	ND	ND	0.02 ± 0.00	ND
Protocatechualdehyde	ND	ND	ND	0.01 ± 0.00	0.01 ± 0.00	ND	ND
Caffeic acid	ND	ND	ND	ND	ND	ND	ND
Rosmarinic acid	ND	ND	ND	ND	ND	ND	ND
Lithospermic acid	ND	ND	ND	0.09 ± 0.00	0.20 ± 0.00	0.29 ± 0.00	0.20 ± 0.01
Salvianolic acid B	0.16 ± 0.01	0.26 ± 0.00	0.27 ± 0.00	0.59 ± 0.02	1.62 ± 0.02	5.34 ± 0.00	2.73 ± 0.02
Salvianolic acid A	ND	ND	ND	ND	ND	0.01 ± 0.00	ND
Salvianolic acid C	0.10 ± 0.00	0.28 ± 0.00	0.28 ± 0.03	0.28 ± 0.04	0.34 ± 0.00	0.33 ± 0.00	0.31 ± 0.00

ND, not detected.

Table S4. Contents of hydrophilic components and moisture in Danshen during drying at 110 ℃ (*n* = 3).

Analyte (mg/g)	Drying time (min)										
	**0**	**10**	**20**	**30**	**40**	**50**	**60**	**70**	**80**	**90**	**100**
Moisture (%)	72.48 ± 0.84	61.37 ± 0.83	47.08 ± 0.51	32.79 ± 4.00	26.42 ± 2.26	22.23 ± 3.09	8.82 ± 1.65	6.72 ± 1.16	4.23 ± 0.63	4.01 ± 0.17	0.66 ± 0.05
Danshensu	0.06 ± 0.00	0.06 ± 0.00	0.06 ± 0.00	0.13 ± 0.00	0.12 ± 0.00	0.10 ± 0.00	0.10 ± 0.00	0.11 ± 0.00	0.12 ± 0.00	0.15 ± 0.00	0.22 ± 0.00
Protocatechuic acid	ND	ND	ND	ND	ND	ND	0.01 ± 0.00	0.03 ± 0.00	0.03 ± 0.00	0.05 ± 0.00	0.07 ± 0.00
Protocatechualdehyde	ND	ND	ND	ND	ND	ND	ND	0.01 ± 0.00	0.01 ± 0.00	0.01 ± 0.00	0.01 ± 0.00
Caffeic acid	ND	ND	ND	0.02 ± 0.00	0.04 ± 0.00	0.04 ± 0.01	0.04 ± 0.00	0.04 ± 0.00	0.05 ± 0.00	0.05 ± 0.00	0.05 ± 0.00
Rosmarinic acid	ND	ND	ND	ND	ND	ND	ND	0.01 ± 0.00	0.06 ± 0.00	0.06 ± 0.00	0.07 ± 0.00
Lithospermic acid	ND	ND	ND	0.23 ± 0.00	0.35 ± 0.00	0.37 ± 0.01	0.39 ± 0.00	0.53 ± 0.00	0.59 ± 0.00	0.62 ± 0.00	0.71 ± 0.00
Salvianolic acid B	0.16 ± 0.01	0.16 ± 0.00	0.98 ± 0.00	2.71 ± 0.05	6.53 ± 0.12	6.87 ± 0.04	6.93 ± 0.11	11.29 ± 0.04	13.73 ± 0.06	14.25 ± 0.16	13.24 ± 0.19
Salvianolic acid A	ND	ND	ND	0.02 ± 0.00	0.03 ± 0.00	0.02 ± 0.00	0.03 ± 0.00	0.03 ± 0.00	0.04 ± 0.00	0.03 ± 0.00	0.06 ± 0.00
Salvianolic acid C	0.10 ± 0.00	0.28 ± 0.01	0.28 ± 0.02	0.42 ± 0.00	0.42 ± 0.00	0.40 ± 0.00	0.36 ± 0.00	0.36 ± 0.00	0.37 ± 0.00	0.34 ± 0.00	0.31 ± 0.00

ND, not detected.

Table S5. Contents of hydrophilic components and moisture in Danshen during drying at 120 ℃ (*n* = 3).

**Analyte (mg/g)**	**Drying time (min)**										
	**0**	**10**	**20**	**30**	**40**	**50**	**60**	**70**	**80**	**90**	**100**
Moisture (%)	72.48 ± 0.84	58.66 ± 1.73	39.89 ± 3.71	17.27 ± 1.81	13.49 ± 4.56	12.22 ± 2.72	4.68 ± 0.72	2.37 ± 0.36	0	0	0
Danshensu	0.06 ± 0.00	0.08 ± 0.00	0.08 ± 0.00	0.09 ± 0.00	0.10 ± 0.00	0.11 ± 0.00	0.12 ± 0.00	0.22 ± 0.00	0.29 ± 0.00	0.41 ± 0.00	0.47 ± 0.00
Protocatechuic acid	ND	ND	ND	0.03 ± 0.00	0.03 ± 0.00	0.03 ± 0.00	0.03 ± 0.00	0.09 ± 0.00	0.09 ± 0.00	0.12 ± 0.00	0.11 ± 0.00
Protocatechualdehyde	ND	ND	ND	0.01 ± 0.00	0.01 ± 0.00	0.01 ± 0.00	0.01 ± 0.00	0.01 ± 0.00	0.01 ± 0.00	0.01 ± 0.00	0.015 ± 0.00
Caffeic acid	ND	ND	ND	0.09 ± 0.00	0.08 ± 0.00	0.09 ± 0.00	0.10 ± 0.00	0.09 ± 0.00	0.07 ± 0.00	0.08 ± 0.00	0.09 ± 0.00
Rosmarinic acid	ND	ND	ND	ND	0.02 ± 0.00	0.02 ± 0.00	0.66 ± 0.01	0.34 ± 0.01	0.35 ± 0.00	0.34 ± 0.00	0.25 ± 0.00
Lithospermic acid	ND	0.08 ± 0.00	0.09 ± 0.00	0.53 ± 0.00	0.51 ± 0.01	0.49 ± 0.00	0.66 ± 0.01	0.87 ± 0.00	0.74 ± 0.00	0.86 ± 0.00	0.82 ± 0.01
Salvianolic acid B	0.16 ± 0.01	1.26 ± 0.00	1.46 ± 0.02	10.67 ± 0.11	10.15 ± 0.12	10.11 ± 0.08	14.25 ± 0.08	21.76 ± 0.07	21.74 ± 0.07	21.37 ± 0.10	19.72 ± 0.10
Salvianolic acid A	ND	ND	ND	0.03 ± 0.00	0.08 ± 0.00	0.09 ± 0.01	0.09 ± 0.00	0.10 ± 0.01	0.09 ± 0.00	0.11 ± 0.00	0.10 ± 0.00
Salvianolic acid C	0.10 ± 0.00	0.10 ± 0.00	0.10 ± 0.00	0.19 ± 0.00	0.25 ± 0.00	0.25 ± 0.00	0.24 ± 0.00	0.22 ± 0.00	0.21 ± 0.00	0.17 ± 0.00	0.25 ± 0.00

ND, not detected.

Table S6. Contents of hydrophilic components and moisture in Danshen during drying at 130 ℃ (n = 3).

**Analyte (mg/g)**	**Drying time (min)**										
	**0**	**10**	**20**	**30**	**40**	**50**	**60**	**70**	**80**	**90**	**100**
Moisture (%)	72.48 ± 0.84	54.73 ± 1.84	28.29 ± 2.71	12.39 ± 2.12	4.12 ± 0.51	4.08 ± 0.69	0.78 ± 0.05	0	0	0	0
Danshensu	0.06 ± 0.00	0.20 ± 0.00	0.11 ± 0.00	0.19 ± 0.00	0.31 ± 0.00	0.32 ± 0.00	0.37 ± 0.00	0.62 ± 0.02	0.90 ± 0.00	1.14 ± 0.00	1.11 ± 0.03
Protocatechuic acid	ND	ND	ND	0.07 ± 0.00	0.08 ± 0.00	0.09 ± 0.00	0.13 ± 0.01	0.13 ± 0.00	0.12 ± 0.00	0.12 ± 0.00	0.11 ± 0.00
Protocatechualdehyde	ND	ND	ND	0.01 ± 0.00	0.01 ± 0.00	0.01 ± 0.00	0.02 ± 0.00	0.02 ± 0.01	0.01 ± 0.00	0.02 ± 0.00	0.01 ± 0.00
Caffeic acid	ND	ND	0.04 ± 0.00	0.08 ± 0.00	0.05 ± 0.00	0.05 ± 0.00	0.07 ± 0.00	0.05 ± 0.01	0.04 ± 0.00	0.04 ± 0.01	0.04 ± 0.00
Rosmarinic acid	ND	ND	0.23 ± 0.00	0.33 ± 0.00	0.32 ± 0.00	0.38 ± 0.01	0.55 ± 0.00	0.70 ± 0.011	0.78 ± 0.00	0.81 ± 0.00	0.79 ± 0.00
Lithospermic acid	ND	0.05 ± 0.00	0.77 ± 0.00	0.95 ± 0.00	0.96 ± 0.00	0.94 ± 0.04	1.08 ± 0.00	1.04 ± 0.02	1.04 ± 0.026	1.23 ± 0.00	1.01 ± 0.01
Salvianolic acid B	0.16 ± 0.01	0.98 ± 0.01	21.65 ± 0.08	21.37 ± 0.24	21.11 ± 0.15	21.69 ± 0.23	27.89 ± 0.17	27.53 ± 0.42	34.89 ± 0.23	27.52 ± 0.16	25.32 ± 0.21
Salvianolic acid A	ND	ND	0.09 ± 0.00	0.14 ± 0.00	0.11 ± 0.00	0.12 ± 0.00	0.17 ± 0.00	0.21 ± 0.00	0.21 ± 0.00	0.22 ± 0.00	0.21 ± 0.00
Salvianolic acid C	0.10 ± 0.00	0.07 ± 0.00	0.30 ± 0.00	0.26 ± 0.01	0.19 ± 0.00	0.18 ± 0.01	0.17 ± 0.00	0.13 ± 0.00	0.12 ± 0.00	0.10 ± 0.00	0.10 ± 0.00

ND, not detected·

Table S7. Contents of hydrophilic components and moisture in Danshen during drying at 140 ℃ (*n* = 3).

**Analyte (mg/g)**	**Drying time (min)**						
	**0**	**10**	**20**	**30**	**40**	**50**	**60**
Moisture (%)	72.48 ± 0.84	49.52 ± 0.89	8.61 ± 1.88	0.96 ± 0.08	0	0	0
Danshensu	0.06 ± 0.00	0.14 ± 0.01	0.14 ± 0.02	0.39 ± 0.01	0.72 ± 0.01	1.90 ± 0.01	1.83 ± 0.09
Protocatechuic acid	ND	MA	0.03 ± 0.00	0.07 ± 0.00	0.08 ± 0.00	0.15 ± 0.00	0.12 ± 0.01
Protocatechualdehyde	ND	MA	0.01 ± 0.00	0.01 ± 0.00	0.01 ± 0.00	0.01 ± 0.00	0.02 ± 0.00
Caffeic acid	ND	MA	0.05 ± 0.00	0.06 ± 0.00	0.04 ± 0.00	0.03 ± 0.00	0.03 ± 0.01
Rosmarinic acid	ND	MA	0.49 ± 0.00	1.28 ± 0.01	1.53 ± 0.02	1.04 ± 0.04	1.12 ± 0.00
Lithospermic acid	ND	0.11 ± 0.00	0.45 ± 0.00	0.58 ± 0.00	0.56 ± 0.00	0.99 ± 0.03	1.10 ± 0.03
Salvianolic acid B	0.16 ± 0.01	0.87 ± 0.01	19.69 ± 0.11	28.38 ± 0.68	28.31 ± 0.99	30.81 ± 0.84	28.48 ± 0.81
Salvianolic acid A	ND	0.04 ± 0.00	0.10 ± 0.00	0.21 ± 0.00	0.23 ± 0.01	0.26 ± 0.00	0.27 ± 0.00
Salvianolic acid C	0.10 ± 0.00	0.14 ± 0.01	0.18 ± 0.00	0.14 ± 0.00	0.12 ± 0.00	0.10 ± 0.00	0.11 ± 0.00

ND, not detected.

Table S8. Contents of hydrophilic components and moisture in Danshen during drying at 150 ℃ (n = 3).

**Analyte (mg/g)**	**Drying time (min)**				
	**0**	**10**	**20**	**30**	**40**
Moisture (%)	72.48 ± 0.84	45.70 ± 0.67	8.04 ± 2.38	0.59 ± 0.81	0
Danshensu	0.06 ± 0.00	0.14 ± 0.00	0.28 ± 0.00	1.56 ± 0.00	2.16 ± 0.02
Protocatechuic acid	ND	ND	0.11 ± 0.01	0.31 ± 0.00	0.32 ± 0.01
Protocatechualdehyde	ND	ND	0.01 ± 0.00	0.02 ± 0.00	0.01 ± 0.00
Caffeic acid	ND	ND	0.03 ± 0.00	0.02 ± 0.00	0.02 ± 0.00
Rosmarinic acid	ND	ND	0.51 ± 0.00	1.36 ± 0.01	1.22 ± 0.00
Lithospermic acid	ND	0.07 ± 0.00	0.85 ± 0.01	1.07 ± 0.00	0.94 ± 0.00
Salvianolic acid B	0.16 ± 0.01	0.36 ± 0.00	29.04 ± 0.15	31.45 ± 0.19	30.82 ± 0.14
Salvianolic acid A	ND	ND	0.14 ± 0.00	0.31 ± 0.00	0.29 ± 0.00
Salvianolic acid C	0.10 ± 0.00	0.22 ± 0.00	0.15 ± 0.00	0.11 ± 0.00	0.07 ± 0.00

ND, not detected.

Table S9. The regression equations of nonlinear quadratic polynomial model at 60–150 ℃.

**Temperature (℃)**	**Drying curve**	R^2
60	$Y = 0.0006X^2 – 0.4160x + 75.246$	0.988
80	$Y = 0.0023X^2 – 0.8480x + 76.983$	0.977
100	$Y = 0.0049X^2 – 1.2128x + 76.860$	0.946
110	$Y = 0.0077X^2 – 1.4863x + 73.404$	0.993
120	$Y = 0.0165X^2 – 2.1637x + 74.492$	0.979
130	$Y = 0.0261X^2 – 2.7843x + 74.823$	0.989
140	$Y = 0.0383X^2 – 3.7030x + 75.041$	0.962
150	$Y = 0.0483X^2 – 3.9831x + 75.535$	0.975

Table S10. Contents of salvianolic acids in Danshen during the drying process at 130 ℃.

**Analytes**	**Drying time (min)**						
	**0**	**20**	**40**	**60**	**80**	**100**	**120**
Moisture (%)	65.14 ± 0.57	61.19 ± 0.24	55.34 ± 0.64	47.17 ± 0.66	24.03 ± 0.38	11.11 ± 0.39	4.79 ± 0.06
Danshensu [a]	ND	ND	ND	0.09 ± 0.00	0.29 ± 0.00	0.58 ± 0.00	0.49 ± 0.01
Protocatechuic acid [a]	ND	ND	ND	ND	ND	0.09 ± 0.00	0.09 ± 0.01
Protocatechualdehyde [a]	ND	ND	ND	0.06 ± 0.00	0.09 ± 0.00	0.13 ± 0.00	0.11 ± 0.00
Caffeic acid [a]	ND	ND	ND	0.09 ± 0.01	0.10 ± 0.00	0.15 ± 0.00	0.10 ± 0.00
Rosmarinic acid [a]	ND	ND	0.24 ± 0.02	0.40 ± 0.00	0.58 ± 0.00	0.73 ± 0.01	0.52 ± 0.02
Lithospermic acid [a]	ND	ND	0.28 ± 0.01	0.48 ± 0.01	0.64 ± 0.02	0.82 ± 0.00	0.61 ± 0.01
Salvianolic acid B [a]	0.17 ± 0.05	8.01 ± 0.68	25.92 ± 0.38	43.02 ± 0.77	55.09 ± 0.60	66.37 ± 0.12	46.11 ± 0.07
Salvianolic acid A [a]	ND	ND	ND	ND	0.10 ± 0.01	0.29 ± 0.01	0.33 ± 0.02
Salvianolic acid C [a]	ND	1.27 ± 0.02	0.76 ± 0.01	0.68 ± 0.02	0.49 ± 0.01	0.62 ± 0.01	0.40 ± 0.01
Total salvianolic acids [a]	0.17	9.28	27.2	44.79	57.38	69.78	48.76
Total phenolic acids [a]	2.01 ± 0.03	11.37 ± 0.30	29.35 ± 0.85	44.97 ± 0.08	66.13 ± 1.37	73.48 ± 1.35	78.99 ± 1.15

[a] the unit: mg/g.

Table S11. DPPH Radical scavenging ability of Danshen samples during the drying process at 130 ℃.

**Samples [a] (mg/mL)**	**Radical scavenging ability (%)**						
	**0 min**	**20 min**	**40 min**	**60 min**	**80 min**	**100 min**	**120 min**
4.4	30.53 ± 1.04	56.40 ± 4.35	83.48 ± 1.24	86.68 ± 0.78	89.73 ± 0.22	90.25 ± 1.05	91.67 ± 0.34
2.2	20.73 ± 1.16	34.52 ± 5.12	71.50 ± 6.54	85.19 ± 0.93	85.12 ± 0.68	90.18 ± 1.61	90.85 ± 0.67
1.1	8.40 ± 1.64	17.56 ± 3.56	37.80 ± 3.47	80.06 ± 1.31	63.69 ± 1.23	90.10 ± 0.90	90.48 ± 0.46
0.55	6.58 ± 0.53	10.79 ± 3.85	22.32 ± 2.57	63.69 ± 1.23	61.68 ± 0.56	87.43 ± 3.23	88.84 ± 1.36
0.28	4.90 ± 0.44	3.20 ± 0.68	10.49 ± 1.02	29.02 ± 1.24	48.51 ± 2.00	48.86 ± 5.79	66.07 ± 2.13
0.14	3.01 ± 1.71	1.93 ± 0.26	2.31 ± 0.13	11.53 ± 0.78	22.25 ± 2.97	22.47 ± 2.37	17.56 ± 3.31
[a]IC_{50}	7.75 ± 0.04	4.08 ± 0.12	1.61 ± 0.06	0.44 ± 0.01	0.43 ± 0.01	0.31 ± 0.02	0.23 ± 0.01

[a] Samples were extracted from dried Danshen root powder by the 70% aqueous methanol and then diluted to the indicated concentration.

Table S12. Lipid peroxidation inhibition activity of Danshen samples during the drying process at 130 ℃.

**Samples** [a] **(**mg/mL**)**	**Lipid peroxidation inhibition activity (%)**						
	**0 min**	**20 min**	**40 min**	**60 min**	**80 min**	**100 min**	**120 min**
4.4	48.80 ± 1.43	63.26 ± 1.16	76.36 ± 1.21	64.90 ± 3.31	76.77 ± 2.44	84.09 ± 4.61	75.51 ± 2.80
2.2	32.27 ± 1.50	55.65 ± 0.32	51.72 ± 3.05	45.36 ± 2.98	48.74 ± 0.44	56.06 ± 3.03	55.44 ± 2.33
1.1	25.11 ± 2.31	27.44 ± 2.63	31.31 ± 1.53	24.50 ± 3.97	23.48 ± 0.76	23.86 ± 1.14	39.93 ± 1.29
0.55	22.77 ± 1.24	17.05 ± 1.47	18.99 ± 1.53	15.23 ± 0.66	20.96 ± 1.16	20.45 ± 3.03	28.49 ± 3.68
0.28	21.41 ± 1.06	8.98 ± 2.87	13.54 ± 1.95	8.61 ± 0.66	10.86 ± 1.91	18.43 ± 0.44	10.60 ± 1.16
0.14	2.63 ± 1.08	3.37 ± 0.84	7.27 ± 0.61	6.62 ± 1.15	6.31 ± 1.58	17.42 ± 0.76	5.82 ± 1.96
[a]IC_{50}	4.85 ± 0.14	3.01 ± 0.05	2.60 ± 0.02	3.20 ± 0.12	2.69 ± 0.08	2.28 ± 0.08	2.24 ± 0.05

[a] Samples were extracted from dried Danshen root powder by the 70% aqueous methanol and then diluted to the indicated concentration.

Table S13. •OH Radical scavenging ability of Danshen samples during the drying process at 130 ℃.

**Samples** [a] **(**mg/mL**)**	**Radical scavenging ability (%)**						
	**0 min**	**20 min**	**40 min**	**60 min**	**80 min**	**100 min**	**120 min**
4.4	62.12 ± 0.71	68.33 ± 1.01	80.24 ± 1.01	73.33 ± 1.35	85.00 ± 2.61	85.95 ± 5.05	82.38 ± 0.67
2.2	37.63 ± 1.07	55.22 ± 0.34	58.33 ± 1.01	65.48 ± 1.01	66.05 ± 3.35	70.95 ± 0.67	72.14 ± 1.68
1.1	27.27 ± 3.57	42.86 ± 6.06	30.95 ± 2.69	41.19 ± 3.70	40.00 ± 0.00	51.67 ± 1.68	54.52 ± 0.34
0.55	14.39 ± 1.79	37.62 ± 4.04	25.71 ± 2.02	31.19 ± 1.01	33.95 ± 5.58	38.81 ± 2.36	37.86 ± 2.36
0.28	12.37 ± 0.36	26.90 ± 3.70	16.43 ± 1.01	23.10 ± 3.03	15.00 ± 1.12	27.38 ± 1.01	27.38 ± 0.34
0.14	9.85 ± 0.36	23.57 ± 1.01	10.48 ± 0.67	19.29 ± 0.34	9.74 ± 3.35	25.00 ± 1.01	23.10 ± 3.70
[a]IC_{50}	2.57 ± 0.03	2.36 ± 0.02	2.27 ± 0.09	1.49 ± 0.08	1.13 ± 0.02	0.74 ± 0.06	0.80 ± 0.05

[a] Samples were extracted from dried Danshen root powder by the 70% aqueous methanol and then diluted to the indicated concentration.

Table S14. O_2^- Radical scavenging ability of Danshen samples during the drying process at 130 ℃.

**Samples** [a] **(**mg/mL**)**	**Radical scavenging ability (%)**						
	**0 min**	**20 min**	**40 min**	**60 min**	**80 min**	**100 min**	**120 min**
4.4	14.16 ± 1.61	22.49 ± 1.74	45.40 ± 0.29	71.57 ± 0.58	70.96 ± 0.29	70.96 ± 0.87	68.71 ± 1.16
2.2	6.51 ± 0.16	16.16 ± 0.58	32.92 ± 0.29	66.16 ± 2.17	69.33 ± 0.58	69.33 ± 1.16	66.97 ± 2.46
1.1	4.22 ± 0.16	10.22 ± 0.58	15.75 ± 3.47	47.03 ± 4.05	50.20 ± 0.14	66.97 ± 1.30	62.88 ± 3.90
0.55	3.88 ± 0.97	10.02 ± 0.58	13.19 ± 6.22	28.63 ± 0.58	43.05 ± 1.59	39.16 ± 1.01	39.16 ± 1.01
0.28	2.85 ± 0.16	7.36 ± 0.58	7.16 ± 2.31	11.76 ± 0.14	17.69 ± 0.72	17.79 ± 1.74	8.79 ± 3.47
0.14	0.68 ± 0.97	5.52 ± 2.02	5.32 ± 0.87	4.70 ± 2.31	7.26 ± 2.46	7.57 ± 2.89	5.52 ± 2.02
[a]IC_{50}	17.25 ± 1.29	14.12 ± 0.08	5.16 ± 0.25	1.35 ± 0.01	1.10 ± 0.04	0.80 ± 0.02	0.86 ± 0.04

[a] Samples were extracted from dried Danshen root powder by the 70% aqueous methanol and then diluted to the indicated concentration.
